# Localized IgG4-related disease manifested on the tongue: a case report

**DOI:** 10.48101/ujms.v126.6118

**Published:** 2021-05-12

**Authors:** Helya Hashemi, Andreas Thor, Erik Hellbacher, Marie Carlson, Miklós Gulyás, Lena Blomstrand

**Affiliations:** a Department of Surgical Sciences, Plastic & Oral and Maxillofacial Surgery, Uppsala University, Uppsala, Sweden; b Department of Medical Sciences, Section of Rheumatology, Uppsala University, Uppsala, Sweden; c Department of Medical Sciences, Gastroenterology Research Group, Uppsala University, Uppsala, Sweden; d Division of Pathology, Department of Immunology, Genetics and Pathology, Uppsala University, Uppsala, Sweden

**Keywords:** IgG4-related disease, localized, oral lesion, tongue, differential diagnosis, histopathology, monoclonal antibody, Rituximab

## Abstract

Immunoglobulin G4-related disease (IgG4-RD) is an immune-mediated fibroinflammatory condition that can affect multiple organs. IgG4-RD may show a variety of initial symptoms. In the oral mucosa, lesions present as inflammatory fibrosis with a large number of IgG4-positive plasma cells. Evaluating treatment is a well-known problem in IgG4-RD due to the absence of an established assessment system. There are difficulties in defining the severity of the disease, which is why treatment is primarily based on its clinical manifestations.

We present a case report of localized IgG4-RD with ulcerative and proliferative manifestations on the tongue, which clinically mimicked oral squamous cell carcinoma. A tumor-like lesion on the tongue can indicate something else other than the malignant or reactive changes commonly found in the oral mucosa. Multiple differential diagnoses of these atypical oral lesions, including localized IgG4-RD, should be considered.

## Introduction

Immunoglobulin G4-related disease (IgG4-RD) is an immune-mediated fibroinflammatory condition affecting multiple organs, which may be difficult to distinguish from other malignant, infectious, and inflammatory diseases. IgG4-RD is characterized by organ enlargement, elevation of serum IgG4 levels, tissue infiltration with IgG4-positive plasma cells and various degrees of fibrosis (1–4). Manifestations have been observed in multiple organ systems, such as the pancreas (5), bile duct (6), retroperitoneum (7), lungs (8), and kidneys (9). In the head and neck region, the common sites of involvement are the major salivary glands, the thyroid, and orbit (10–18).

In this report, we present a case report of localized IgG4-RD with ulcerative and proliferative manifestations on the tongue, which clinically mimicked oral squamous cell carcinoma. After excluding multiple diagnoses, we considered localized IgG4-RD to be the likely diagnosis. We present the protracted clinical manifestations lasting for 5 years, diagnostic work-up, pathology, and management of this case.

## Case report

**Figure 1:**
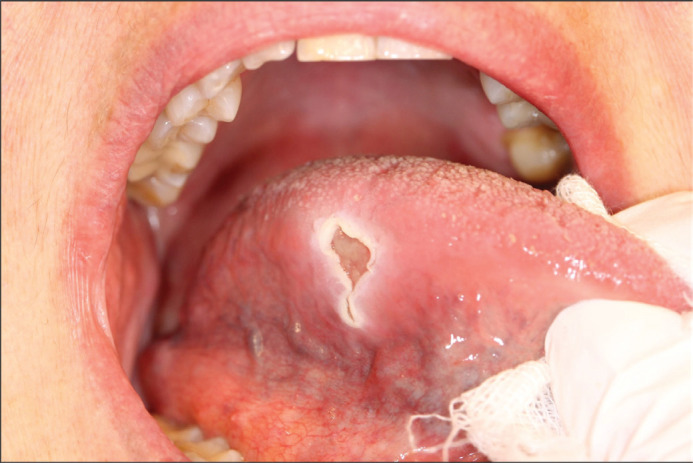
First visit in December 2014 showing a firm ulcer with a fibrinous base.

In December 2014, a 71-year-old woman was referred to the Department of Plastic and Oral and Maxillofacial surgery at Uppsala University from the Ear, Nose, and Throat Department (ENT). The reason stated for referral was a painful ulcer on the right side of the tongue, which had manifested 6 months earlier. The ulcer had a fibrinous base and was firm, but not indurated (Figure 1). There were no lymphadenopathies nor a history of fever. The patient was a non-user of tobacco but consumed moderate amounts of alcohol. She had a history of type 2 diabetes, hypertension, asthma, and osteoporosis. Intake of medications included Montelukast, Budesonide, Salbutamol, Prednisolone, Acetylcysteine, Omeprazole, Colestipol, Loperamide, Felodipine, Furosemide, Losartan, Zopiclone, and Alendronic acid. An incisional biopsy with histopathological examination (HPE) was conducted from the lateral border of the tongue. It displayed an inflamed tongue mucosa, with the epithelium being hyperplastic and hyperkeratinized. Blood tests showed C-reactive protein levels of 11 mg/L (< 5 mg/L) and a white blood cell count of 10.8 (3.5–9–10^9^ cells/L). There were no signs of dysplasia, and malignancy could be ruled out. She received treatment with Clobetasol and Chlortetracycline with no improvement of the symptoms.

In January 2015, the lesion had increased in size, and an excisional biopsy was performed. The initial diagnostic hypothesis was a drug-induced side effect initiated by the Alendronate treatment (19, 20) for osteoporosis, which caused an eosinophilic ulcer (21, 22). A computed tomography (CT) scan did not show any lesions of the mandible or signs indicative of bisphosphonate-related osteonecrosis of the jaw. An HPE of the tongue showed an ulcer affected with oral candidiasis. The patient was treated with antimycotics. Alendronate therapy was ceased and replaced with Prolia (Denosumab).

**Figure 2:**
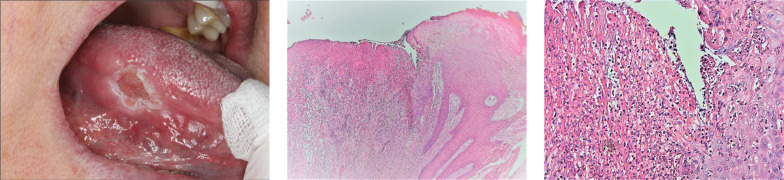
(a) In June 2015 an increase in size of the ulcer was seen and an excision was performed. (b) & (c) Micrographs showing ulceration with fibrinous crusta and infiltrate of neutrophil granulocytes. Hematoxyline and eosin staining at × 40 (2b) and × 400 (2c).

**Figure 3:**
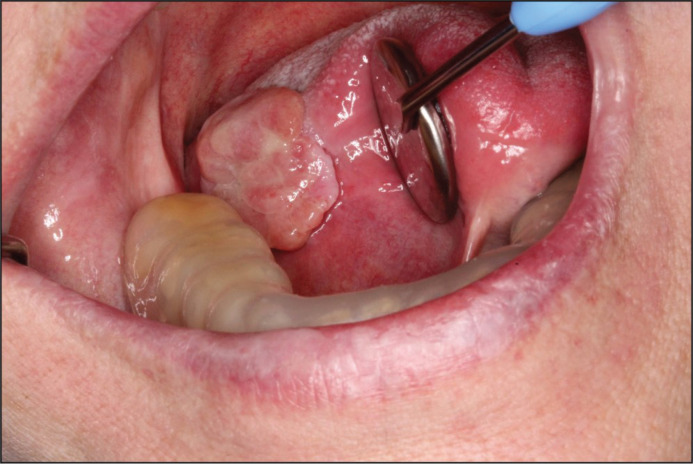
In December 2015 the lesion had an exophytic appearance. A dental mouthguard can be seen covering the teeth of the lower jaw to protect the mucosa of the tongue.

**Figure 4:**
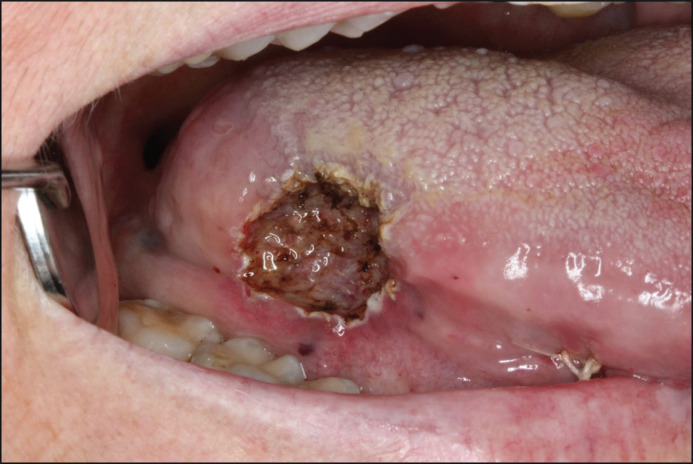
Treatment with radiofrequency ablation of the tongue performed in February 2016.

In June 2015, the patient reported increased pain from the enlarged ulcer without improvement of symptoms. A new excision was performed (Figure 2a), where HPE did not show any signs of dysplasia, but revealed an ulceration with fibrinous crusta, inflammation, and extensive infiltrates of neutrophil granulocytes (Figure 2b and c). At a revisit in September 2015, there was an ulcer with fungal infection. Fluconazole was administered, and the patient was called in for re-examination in December 2015. At that point, the lesion had developed an exophytic appearance on the lateral side of the tongue (Figure 3). In February 2016, treatment with radiofrequency ablation of the tongue was carried out (Figure 4). HPE displayed moderately to severely inflamed mucosa, with fibrosis and an ulcer consisting of eosinophil-rich infiltrates with candida infection. There were still no signs of malignancy or dysplasia. Antimycotics were administered.

**Figure 5:**
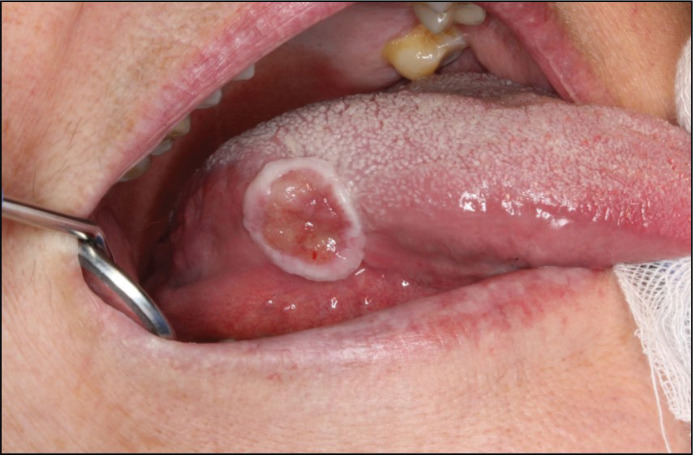
Follow-up in April 2016: a reoccurring exophytic growth of the lesion could be observed.

At the follow-up in April 2016, there was a recurrent exophytic growth from the lesion (Figure 5). Simultaneously, a gastroenterologist examined the patient further regarding her diffuse form of colitis and eosinophil involvement in the intestine, suspecting eosinophilic colitis. The patient also showed elevated levels of serum eosinophil cationic protein 24 µg**/**L (2.3–16 µg**/**L) during the examination, and the possible diagnosis of hyper eosinophilic syndrome (23) was contemplated. For this reason, the patient underwent detection for molecular abnormality of dysregulated tyrosine kinase activity. Fluorescence *in situ* hybridization was used to detect the presence of FIP1L1-PDGFRA in bone marrow cells in order to further determine the treatment approach (24, 25).

Daily treatment with 10 mg × 3 Prednisolone was initiated. The patient reported noticeable improvement regarding pain-related symptoms both from the oral cavity and from the stomach. However, there was no regress of the lesion. A possible differential diagnosis of Pyostomatitis vegetans (PV) associated with inflammatory bowel disease was considered. As the patient had not displayed any characteristic pustules or a confirmed diagnosis of ulcerative colitis or Crohn’s disease, and had not shown any improvement when treated with Prednisolone, PV was excluded as a possible diagnosis.

**Figures 6 & 7:**
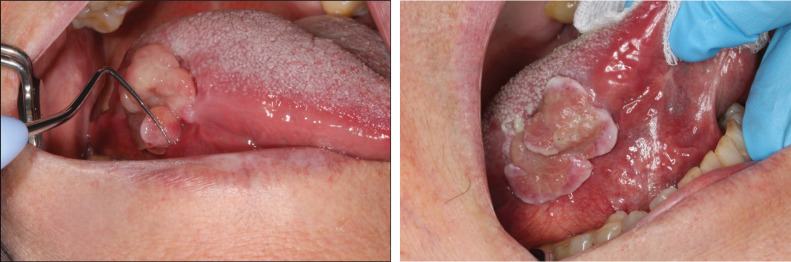
During 2016 and 2017, due to several recurring exophytic growths of the lesion, the patient underwent multiple excisions.

During the period 2016 and 2017, due to several recurring exophytic growths of the lesion, the patient underwent multiple excisions (Figures 6 and 7). We concluded that surgical treatment was unsuitable for this specific lesion. An HPE consistently displayed mild-to-severe inflammation, without signs of dysplasia. To exclude malignancy, detection of Epstein–Barr virus was carried out. The results were negative (26).

In February 2018, our patient was investigated at the Department of Rheumatology. Churg–Strauss syndrome (CSS), now better known as eosinophilic granulomatosis with polyangiitis (EGPA), was discussed as a possible differential diagnosis. EGPA is a form of vasculitis, affecting small- and medium-sized arteries. It causes granulomatous, eosinophilic inflammations, mostly found in patients with severe asthma (27). Overall, the clinical picture, signs, and symptoms were not considered typical for EGPA. It was at this point that the suspicion of IgG4-RD was raised.

**Figure 8:**
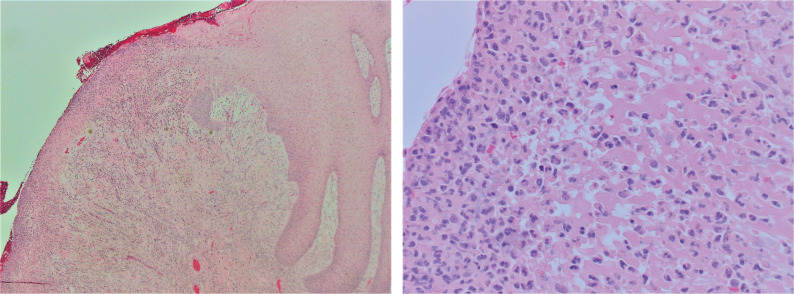
(a) & (b) Micrographs showing ulceration with extruding granulation tissue on the left, and normal epithelial surface with slight lymphoplasmacytic infiltrate underneath on the right. Hematoxyline and Eosin staining at x 40 (8a) and x 400 (8b).

Furthermore, in February 2018, another excision biopsy was retrieved from the lesion on the tongue, with an inquiry regarding IgG4-positive plasma cell levels. Histopathological analysis revealed that the tumor-like lesion on the tongue was caused by a candidiasis-associated ulceration. The ulceration formed an extruding polyploid lesion with granulation tissue rich in neutrophil granulocytes and lymphoplasmacytic cells (Figure 8a and b). The presence of plasma cells was demonstrated by CD138 and CD38 immunostainings. Eosinophils could be seen; however, there were no signs of granuloma or vasculitis. IgG-positive plasma cells were observed a few millimeters from the ulceration, further strengthening the suspicion of IgG4-RD (28, 29).

**Figure 9:**
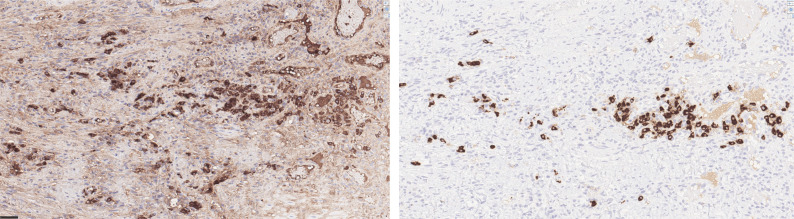
(a) & (b) Immunostainings for IgG (9a) and IgG4 (9b) in area under retained epithelium, both at x 400. Majority of IgG-positive plasma cells shows positive staining for IgG4.

An IgG-immunostaining was performed, a method that can be unreliable and difficult to interpret due to high-background staining (Figure 9a). As a result, accurate IgG4/IgG ratios are sometimes difficult to obtain (30, 31). In our case, the IgG-immunostaining was performed on the two larger biopsies. In both samples, ≥ 71% of the IgG-positive plasma cells expressed IgG4 (Figure 9b), that is, an IgG4+/IgG+ ratio of ≥ 71%, with IgG4+ plasma cells/high-power field (HPF) being 10–50. While a significant percentage of the plasma cells expressed IgG4, the patient did not have elevated levels of IgG4 in the serum. However, this parameter is not precise enough and lacks a predictive value for diagnosis (31, 32).

Medical databases and literature revealed only one description of IgG4-RD involving the tongue (33). Therefore, two hypotheses were discussed: one being a possible fibro-inflammatory disease called IgG4-RD, with an unknown etiology (34), and the other one being Circumoroficial plasmacytosis (35). We believed the latter hypothesis to be less likely because of the presence of a large number of IgG4-positive plasma cells.

A more conclusive diagnosis of IgG4-RD would have validated treatment with Rituximab, a monoclonal antibody that targets CD20 presented by B cells (36). With the suspicion of IgG4-RD, treatment was initiated with 20 mg/week of Methotrexate together with a low dose of Prednisolone for 3 months, followed by evaluation. The patient’s symptoms improved but the condition remained.

**Figure 10:**
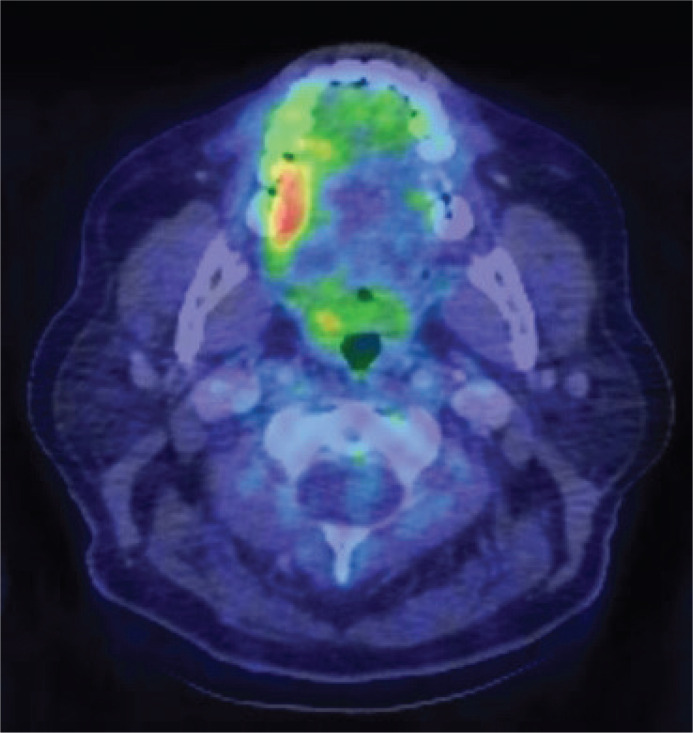
FDG PET/CT showing an increased uptake on the right side of the mouth corresponding to the right side of the tongue and cheek.

In August 2018, 6 months after the latest excision biopsy of the lesion, the patient underwent fluoro-deoxy-glucose positron emission tomography with computerized tomography (FDG PET/CT) for evaluation and diagnosis of IgG4-RD. This method enabled an individually tailored treatment and provided a better understanding of the disease (37). FDG PET/CT showed involvement of multiple groups of lymph nodes in the mediastinum and neck. There was also an increased uptake on the right side of the mouth, directly corresponding to the right side of the tongue and cheek (Figure 10). A neck lymph node biopsy demonstrated elevated levels of IgG4-positive plasma cells, displaying an IgG4+ plasma cells/HPF of 10–50 and an IgG4+/IgG+ ratio of ≥71%. This further strengthened our suspicions of IgG4-RD.

**Figure 11:**
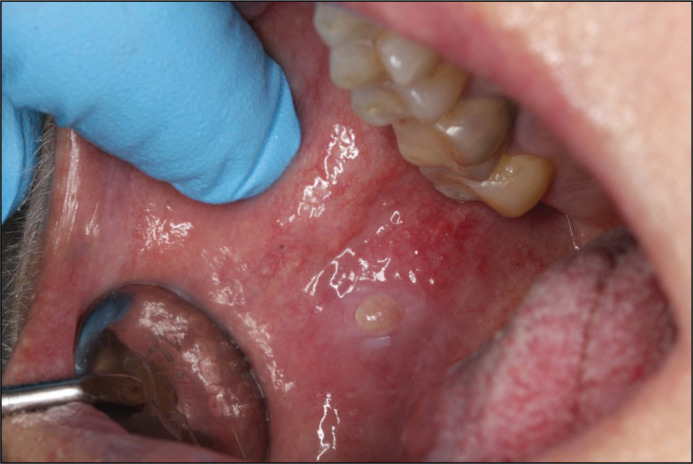
In September of 2018, a new, firm lesion of the right buccal mucosa could be observed.

In September 2018, a follow-up showed a progressive exophytic growth on the tongue. New, firm, soft tissue changes in the mucous membrane on the right side of the buccal cheek, much like the previous changes seen on the tongue, were observed (Figure 11). This new growth was also noticeable on FDG PET/CT in August, a month before. The patient experienced pain mainly on the tongue, and thus, treatment with Rituximab was initiated.

**Figure 12:**
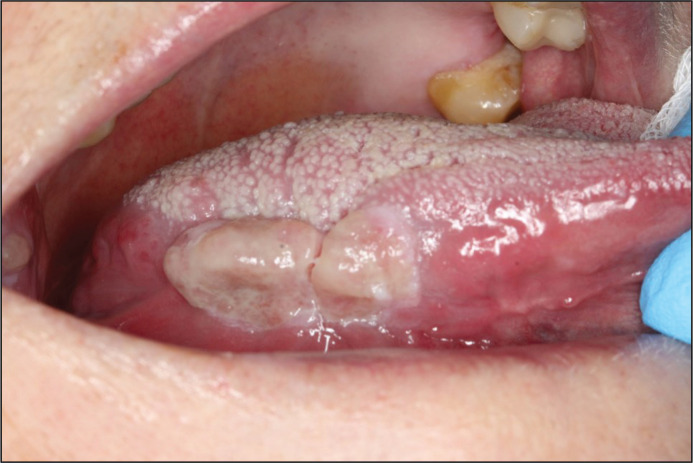
In 2019, the lesion of the tongue remains, however is painless, less firm and without colour change or obvious progression. The lesion of the right buccal mucosa remains unchanged.

Approximately 1 year later, in 2019, the patient reported that she was completely pain-free. The lesions remained but were significantly less firm in consistency. Neither color change nor progression of the lesions were observed (Figure 12).

## Discussion

In 2019, the American College of Rheumatology (ACR) and the European League Against Rheumatism (EULAR) developed new international classification criteria for IgG4-RD. The entry criteria require involvement of IgG4-RD in at least one organ. The exclusion criteria consist of 32 items, all of which must be unfulfilled to reach IgG4-RD classification. Finally, eight weighted inclusion criteria domains are applied, creating a point system that must meet a specific threshold (31). In the head and neck region, there are a few diseases classified as IgG4-RD or manifestations thereof.

Mikulicz’s disease (MD) was first reported in 1888. It is characterized by painless, symmetrical, bilateral, enlarged salivary and lacrimal glands with lymphocytic infiltration. The histological findings of the disease were identical to Sjogren’s syndrome (SS), and MD was, therefore, considered to be a subtype of SS. However, the clinical picture and the histopathology of the two diseases vary, and in MD, one can find elevated levels of serum IgG4, as well as IgG4-positive cell infiltration. Hence, the disease was suggested to represent an systemic IgG4-RD (11, 14, 18).

Küttners tumor (KT), a disorder in the salivary glands associated with IgG4-RD, is an inflammatory disease previously known as chronic sclerosing sialadenitis. It was first described in 1896, and it primarily affects the submandibular glands. Histologically, KT shows fibrosclerotic lesions containing IgG4-positive plasma cells. The histopathological and serological findings in KT are similar to those of MD (38, 39).

There are reports of mucosal manifestations of IgG4-RD in the head and neck region as well. Most of these lesions have occurred on the mucosa of the gingiva, the alveolar mucosa, hard palate, and floor of the mouth. Manifestations on the laryngeal mucosa, nasal and paranasal sinus involvements have also been described (29). These lesions present as inflammatory fibrosis with high amounts of IgG4-positive plasma cells. One of the studies suggested that plasma cell mucositis is not related to IgG4-RD, but IgG4-positive plasma cells are often seen in chronic oral inflammatory conditions (28). As of now, only one case of IgG4-related sclerosing disease involving the tongue has been reported in 2013 (33).

We report a case of localized IgG4-RD manifesting as a progressive tumoral lesion on the lateral border of the tongue. Before reaching the final diagnosis of IgG4-RD and administering adequate treatment, many possible diagnoses were investigated and correspondingly treated without success. Throughout treatment, the mucosal lesion changed in character from a small ulcer to a painful, exophytic tumor-like lesion. With this case, we show that a tumor-like lesion on the tongue can suggest something else other than the malignant or reactive changes commonly found in the oral mucosa. Our case emphasizes the importance of working in a multidisciplinary manner in the absence of expected treatment results. Multiple differential diagnoses of these atypical oral lesions, including localized IgG4-RD, should be considered. More importantly, our case underlines the importance of being attentive when symptoms do not match histopathological findings, and why one should never exclude possible connections to the patient’s underlying conditions.

Evaluating treatment in IgG4-RD is a well-known problem due to the absence of an established assessment system. Difficulties remain in outlining the severity of the disease. Treatment is, therefore, based mostly on the clinical manifestations and not on the severity of the disease. Retrospectively, treatment with Rituximab was identified as successful due to the lack of progression in size and relief of pain from the lesion. Further studies are needed to determine the pathophysiology and epidemiology to efficiently diagnose and treat cases of localized or general IgG4-RD.

Based on the ACR/EURLAR classification criteria for IgG4-RD, our patient met the entry criteria. No exclusion criteria were detected. A threshold of 20 points is needed for a patient to be classified as having IgG4-RD. As obliterative phlebitis and storiform fibrosis were not observed in the excision biopsy of the tongue, 18 of a possible 29 points were obtained from the histopathology criteria.

Furthermore, for the classification criteria for IgG4-RD, no additional points could be obtained for our patient to achieve the threshold of 20 out of a possible 96 points. However, based on the histopathological findings, ruling out other possible diagnoses and the fact that Rituximab was the only successful treatment leading to improvement of the patient’s symptoms, it was concluded that a localized form of IgG4-RD of the tongue was the most likely diagnosis.
